# Single-cell and multi-omics analysis reveals the role of stem cells in prognosis and immunotherapy of lung adenocarcinoma patients

**DOI:** 10.3389/fimmu.2025.1634830

**Published:** 2025-07-22

**Authors:** Jianan Zheng, Haoran Lin, Wei Ye, Mingjun Du, Chenjun Huang, Jun Fan

**Affiliations:** Department of Thoracic Surgery, The First Affiliated Hospital with Nanjing Medical University, Nanjing, China

**Keywords:** lung adenocarcinoma, single-cell sequencing, stem cells, prognostic model, immunotherapy

## Abstract

**Background:**

The roles of stem cells in lung adenocarcinoma (LUAD) progression and therapeutic resistance have been recognized, yet their impact on patient prognosis and immunotherapy response remains unclear.

**Methods:**

Single-cell RNA sequencing was performed to identify stem cell populations characterized by high expression of MKI67 and STMN1. Key marker genes were identified using the FindAllMarkers function, and these genes were subsequently analyzed for mutations, copy number variations, and prognostic significance in LUAD patients. Multiple machine learning algorithms were systematically compared in order to develop an optimal prognostic model. The predictive performance of the model was validated across seven independent LUAD cohorts and immunotherapy datasets. Patterns of immune infiltration were assessed using various computational approaches and were further validated in an internal hospital cohort.

**Results:**

Through comprehensive machine learning optimization, CoxBoost+Enet (alpha=0.7) was identified as the optimal model, incorporating seven key stem cell–related genes and designated as the Stem Cell Prognostic Model (SCPM). Patients were consistently stratified into high- and low-SCPM groups across all seven validation cohorts, with poorer overall survival observed in the high-SCPM group. Predictive accuracy was demonstrated by ROC analysis (AUC > 0.65), while clear group separation was confirmed through PCA based on the seven-gene signature. Notably, immunotherapy response was also predicted by SCPM, with inferior outcomes observed in high-SCPM patients following treatment with immune checkpoint inhibitors. Significantly lower immune cell infiltration, characteristic of “cold” tumors, was detected in high-SCPM patients by multiple immune infiltration algorithms. These findings were further validated in the internal cohort, where reduced CD8+ T cell infiltration was observed in high-SCPM patients.

**Conclusion:**

A stem cell–based prognostic model (SCPM) was constructed and validated, enabling accurate prediction of survival and immunotherapy response in LUAD patients. Patients with immunologically “cold” tumors, as identified by the SCPM, may benefit from alternative therapeutic strategies.

## Introduction

Lung cancer is the leading cause of cancer-related morbidity and mortality worldwide, with over 2.2 million new cases and approximately 1.8 million deaths annually according to the World Health Organization (1, 2). Lung adenocarcinoma (LUAD), as the predominant histological subtype of non-small cell lung cancer, accounts for 40-50% of all lung cancers (3, 4). Despite significant advances in molecular targeted therapy and immunotherapy in recent years, the 5-year survival rate for LUAD patients remains low (5, 6). Tumor heterogeneity, early metastatic tendency, treatment resistance, and disease recurrence continue to be key factors limiting long-term patient survival, creating an urgent need for novel biomarkers to guide precision treatment strategies.

Cancer stem cell (CSC) theory provides a new perspective for understanding tumor heterogeneity and treatment resistance (7–10). Cancer stem cells are a subset of tumor cells with self-renewal capacity and multipotent differentiation potential. They play crucial roles not only in tumor initiation, progression, metastasis, and recurrence but also serve as an important source of tumor heterogeneity (11). In lung cancer, cancer stem cells typically exhibit resistance to chemotherapy and radiotherapy, enabling survival under treatment pressure and causing disease recurrence (12). MKI67, as a classic marker of cell proliferation, has expression levels closely associated with tumor aggressiveness and patient prognosis. STMN1, as an important microtubule-regulating protein, participates in cell cycle regulation and anti-apoptotic processes through microtubule dynamics control, playing a key role in maintaining cancer stem cell properties. The rapid development of single-cell RNA sequencing technology provides powerful tools for in-depth investigation of tumor heterogeneity and cancer stem cell characteristics, enabling researchers to dissect the complexity of the tumor microenvironment at the single-cell level (13).

Immunotherapy, particularly the application of immune checkpoint inhibitors, has brought revolutionary changes to lung cancer treatment (14). Immune checkpoint inhibitors such as PD-1/PD-L1 and CTLA-4 antibodies reactivate the body’s anti-tumor immune response by relieving tumor cell suppression of the immune system (15, 16). However, immunotherapy also faces significant challenges: only approximately 20-30% of LUAD patients achieve durable clinical benefit from immunotherapy, while the majority exhibit primary or acquired resistance (17). The immune cell infiltration status in the tumor microenvironment, particularly the infiltration levels of effector immune cells such as CD8+ T cells and CD4+ T cells, is considered a key factor affecting immunotherapy efficacy. “Hot tumors” typically have abundant immune cell infiltration and respond well to immunotherapy, while “cold tumors” are characterized by sparse immune cell infiltration, leading to poor immunotherapy outcomes. Studies suggest that cancer stem cells may influence the immune status of the tumor microenvironment through mechanisms such as secreting immunosuppressive factors and recruiting regulatory T cells (18).

Based on the above background, this study aims to systematically identify cancer stem cell-like subpopulations in LUAD and deeply analyze their molecular characteristics and biological functions by integrating single-cell RNA sequencing data with large-scale clinical cohort data. We employ advanced machine learning algorithms to construct a Stem Cell Prognostic Model (SCPM) based on stem cell-related genes and validate its predictive performance across multiple independent cohorts. Additionally, we will explore the potential value of this model in predicting immunotherapy response and analyze the relationship between cancer stem cell characteristics and the tumor immune microenvironment. We hypothesize that tumors with high cancer stem cell features exhibit “cold tumor” characteristics and show poor responsiveness to immunotherapy. The findings of this study will provide novel biomarkers and theoretical foundations for precision diagnosis and treatment of LUAD.

## Method

### Dataset source

LUAD gene expression profiles, somatic single nucleotide variants (SNVs), and copy number alterations (CNAs) were obtained from The Cancer Genome Atlas (TCGA) repository, while normal lung tissue expression profiles were retrieved from the Genotype-Tissue Expression (GTEx) database for comparative analysis. Recurrent genomic amplifications and deletions were identified through the application of GISTIC 2.0 algorithm (https://software.broadinstitute.org) to TCGA-derived CNA data. Six independent LUAD datasets were consolidated from the Gene Expression Omnibus (GEO) repository: GSE13213 (19) (n=117), GSE26939 (20) (n=115), GSE29016 (21) (n=39), GSE30219 (22) (n=85), GSE31210 (23) (n=226), and GSE42127 (24) (n=133). Inter-cohort batch effects were eliminated using the ComBat normalization method (25), followed by standard data preprocessing procedures. To assess the model’s performance in immunotherapy contexts, three immunotherapy-associated NSCLC datasets were compiled: POPLAR (26) (n=59), OAK (26) (n=257), SU2C (27)(n=130).

### scRNA-seq data processing

Single-cell transcriptomic data were sourced from GEO: GSE241934. Raw transcriptomic profiles were preprocessed using the Seurat R package (28) (version 4.2.0). Analytical inclusion criteria were established with a minimum expression threshold requiring each gene to be detected in at least 10 cells per sample. Cell quality filtering was performed according to predefined parameters: cells were excluded if they exhibited expression of more than 5000 or fewer than 200 genes, or if mitochondrial genome-derived unique molecular identifiers (UMIs) comprised more than 10% of total counts. Sample integration was achieved through the harmony R package implementation. The analytical workflow encompassed highly variable gene identification for principal component analysis (PCA), followed by dimensionality reduction employing the top 30 principal components via t-distributed Stochastic Neighbor Embedding (t-SNE) methodology. Subpopulation-specific expression signatures were characterized using the “FindAllMarkers” function, with cellular phenotype annotation performed based on established lineage-specific markers from previous studies (29).

### Development of cancer stem cell-derived prognostic signature

Through single-cell transcriptomic profiling, MKI67+STMN1+ cancer stem cell subsets were characterized, with downstream investigations performed utilizing their distinctive molecular markers. Gene expression differences were assessed via the limma software package (30), applying cutoff criteria of FDR < 0.05 and |log2FC| > 1. Mutational profiles of target genes were systematically examined through maftools package implementation. Visual representation of frequently altered stem cell-related genes was achieved using Oncoplot methodology, whereas prevalent mutational signatures were uncovered via signature profiling techniques. Machine learning methodologies serve essential functions in prognostic biomarker construction (31, 32). For developing a reliable Stem Cell Prognostic Model (SCPM), preliminary univariate Cox proportional hazards analysis was executed to detect survival-related genetic elements. The refinement process encompassed thorough assessment of diverse algorithmic strategies via 10-fold cross-validation techniques (33). Multiple computational methodologies were integrated within the analytical framework: sequential Cox modeling, Lasso constraint methods, Ridge penalization, Cox-adapted partial least squares regression (plsRcox), CoxBoost algorithms, random survival forests (RSF), gradient boosting machines (GBM), elastic net (Enet) constraints, supervised principal component (SuperPC) techniques, and survival-oriented support vector machines (survival-SVM). This comprehensive assessment was structured to determine the most effective SCPM architecture, employing concordance index (C-index) as the principal evaluation criterion. Performance validation of the constructed SCPM was accomplished using temporal ROC curve assessment, Kaplan-Meier survival estimation, and principal component analysis (PCA) techniques.

### Immunological profile assessment

Assessment of immunotherapy sensitivity in LUAD patients was conducted using immunophenoscore (IPS) calculations through The Cancer Immunome Atlas database (https://tcia.at/home) (34). Characterization of the tumor immune microenvironment was performed via ssGSEA methodology, which measured immune cell penetration dynamics and immunological pathway activity levels in malignant tissue samples. Detailed immune cell infiltration data from TCGA cohorts were obtained using the TIMER2.0 resource (35), which integrated results from various analytical algorithms.

### Clinical sample acquisition and transcriptomic analysis

Tissue specimen procurement was granted ethical clearance by the Medical Ethics Board of the First Affiliated Hospital of Nanjing Medical University. These specimens were verified as pulmonary adenocarcinoma by pathological specialists, retrieved during surgical procedures, and subsequently transported to Oncocare Inc. (Suzhou, China) for transcriptomic sequencing analysis.

### Study population and specimen acquisition

Tissue specimens consisted of formalin-fixed paraffin-embedded sections obtained from the Pathology Department of The First Affiliated Hospital of Nanjing Medical University. Histopathological confirmation of LUAD diagnosis was established for all samples, with inclusion limited to therapy-naive patients prior to operative procedures. Ethical approval for the investigation was granted by the Institutional Review Board of The First Affiliated Hospital of Nanjing Medical University, and informed consent documentation was secured from each participant. These histological sections were processed for multiplex immunohistochemical examination.

### Multi-marker immunohistochemical staining

The correlation between prognostic scores and immunocyte penetration was assessed through multiplex fluorescent immunolabeling techniques. Specimen preparation commenced with dewaxing in xylene solution, subsequently followed by tissue rehydration using graduated alcohol concentrations. Following epitope unmasking and protein blocking with 5% caprine serum, tissue sections were subjected to consecutive primary antibody incubations: CD4 (dilution 1:500, Cat# ab133616), CD8 (dilution 1:2000, Cat# ab217344), and CD20 (dilution 1:100, Cat# ab64088), after which fluorescent-labeled secondary antibodies were applied. Cell nuclei visualization was accomplished using DAPI counterstain.

### Statistical methods

Data analysis was conducted using R software version 4.2.0. Two-group comparisons were analyzed through unpaired Student’s t-test for parametric data, whereas Mann-Whitney U test was utilized for non-parametric distributions. Multi-group analyses were executed using one-way ANOVA with subsequent Tukey’s multiple comparison testing for parametric data, while Kruskal-Wallis analysis followed by Dunn’s multiple comparison procedure was applied for non-parametric datasets. Pearson’s correlation coefficients were calculated to assess variable relationships. Results were expressed as mean ± standard deviation (SD). The threshold for statistical significance was established at P < 0.05 (*P < 0.05, **P < 0.01, ***P < 0.001).

## Result

### Single-cell RNA sequencing analysis reveals expression patterns and mutational characteristics of stem cell marker genes

**Figure 1:**
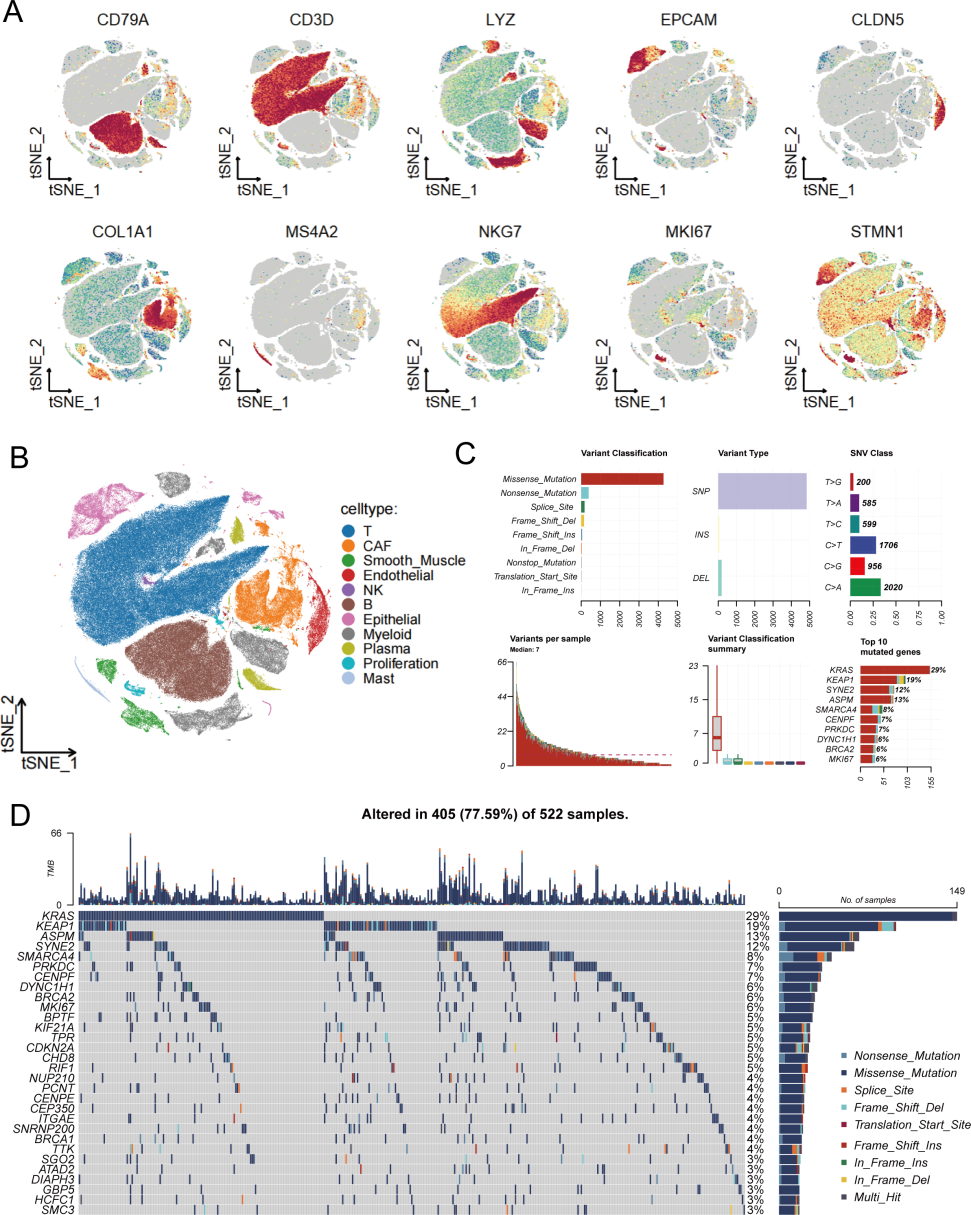
Single-cell transcriptomic analysis and mutational characteristics of stem cell-related genes. **(A)** t-SNE plots showing expression distribution of various cell type-specific marker genes, with MKI67 and STMN1 serving as stem cell markers, where color intensity reflects gene expression levels. **(B)** Cell type annotation based on marker gene expression, identifying various cell populations within the tumor microenvironment to provide cellular context for stem cell research. **(C)** Systematic analysis of stem cell marker gene mutation spectrum, including variant classification distribution (upper left), variant type statistics (upper middle), single nucleotide variant categories (upper right), sample mutation burden distribution (lower left), variant classification summary (lower middle), and ranking of most frequently mutated stem cell marker genes (lower right). **(D)** Mutational landscape waterfall plot of stem cell marker genes, specifically displaying mutation patterns of the top 30 most frequently mutated stem cell marker genes across 522 lung adenocarcinoma samples, with different colors representing different types of genomic alterations, providing direct evidence for understanding the molecular mechanisms of stem cell abnormalities in lung adenocarcinoma pathogenesis.

To deeply explore the role of stem cells in LUAD development and progression, we first analyzed the distribution characteristics of various cell types in the tumor microenvironment using single-cell RNA sequencing technology. Through t-SNE dimensionality reduction visualization (**Figure 1A**), we systematically examined the expression distribution of multiple cell type–specific markers, including B cells (CD79A), T cells (CD3D), myeloid cells (LYZ), epithelial cells (EPCAM), endothelial cells (CLDN5), fibroblasts (COL1A1), mast cells (MS4A2), natural killer cells (NKG7), as well as proliferation-associated stem cell marker genes MKI67 and STMN1. Based on the expression profiles of these marker genes, we successfully identified the major cell types within the tumor microenvironment (**Figure 1B**), providing a foundation for further investigation of stem cell roles in the complex tumor microenvironment. It should be noted that the “proliferative stem cells” defined in this study are a population selected based on high expression of broad proliferation-related markers (MKI67 and STMN1), and actually comprise a mixture of cell subtypes with high proliferative activity, rather than strictly corresponding to classical definitions of cancer stem cells or mesenchymal stem cells. Building upon the comprehensive characterization of the tumor microenvironment’s cellular composition, we further focused on analyzing the mutational characteristics of stem cell marker genes (**Figure 1C**). For the selection of marker genes included in the mutation analysis, we applied the following parameters: only.pos = TRUE, min.pct = 0.25, and logfc.threshold = 0.25. Mutational spectrum analysis targeting stem cell-related genes revealed that missense mutations were the predominant variant type, suggesting these mutations may directly affect stem cell biological properties by altering protein function. Variant type analysis showed that single nucleotide polymorphisms (SNPs) dominated among stem cell marker genes, while insertions (INS) and deletions (DEL) were relatively rare. Single nucleotide variant (SNV) analysis revealed that C>A and C>T transitions were the most common mutation types in stem cell marker genes, potentially related to defects in DNA damage repair mechanisms in stem cells. Analysis of mutational burden distribution across samples revealed significant inter-patient variability in the mutation frequency of these genes. Finally, we constructed a comprehensive mutational landscape of stem cell marker genes (**Figure 1D**), specifically displaying the top 30 most frequently mutated stem cell marker genes across 522 LUAD samples, with 405 samples (77.59%) harboring at least one stem cell-related gene alteration. Among these highly mutated stem cell marker genes, KRAS showed the highest mutation rate (29%). As a key signaling pathway gene regulating stem cell self-renewal and proliferation, KRAS mutations may directly activate the oncogenic transformation capacity of stem cells. KEAP1, as the second most frequently mutated gene, participates in oxidative stress response regulation, and its mutations may enhance the antioxidant capacity and survival advantage of stem cells. SYNE2, the third most frequently mutated gene, primarily participates in nuclear envelope structure maintenance and nuclear morphology regulation, and its mutations may affect nucleocytoplasmic interactions and gene expression regulatory mechanisms in stem cells. This specialized mutational landscape analysis of stem cell marker genes provides important molecular foundations for understanding the molecular aberrant mechanisms of tumor stem cells and developing precise stem cell-targeted therapeutic strategies.

### Comprehensive analysis of stem cell marker gene expression alterations and associated biological pathways

**Figure 2:**
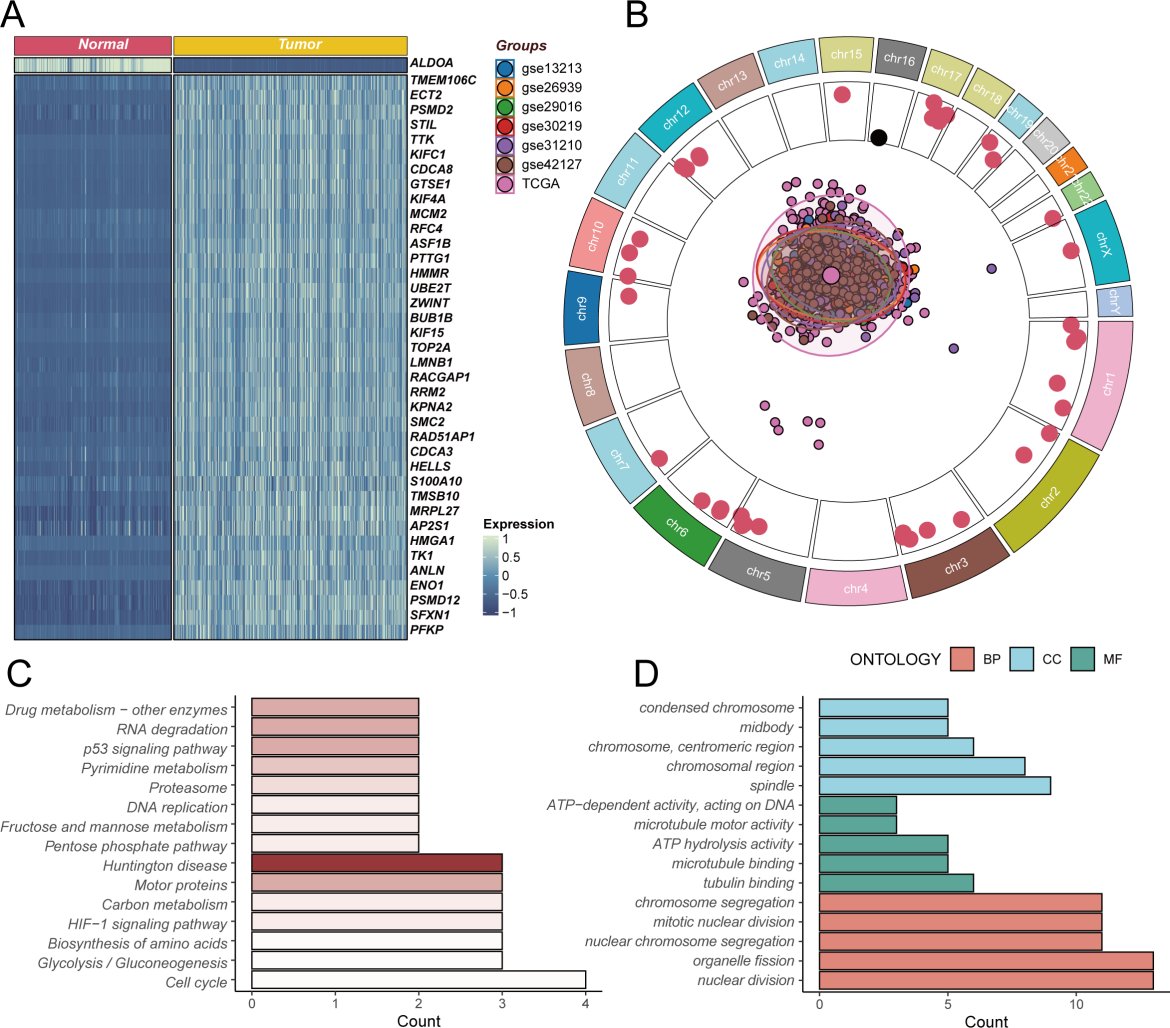
Differential expression analysis and functional enrichment of stem cell marker genes. **(A)** Heatmap of differentially expressed stem cell marker genes based on GTEx normal lung tissues and TCGA lung adenocarcinoma tissues, with blue indicating low expression and red indicating high expression, showing upregulation of TMEM106C, ECT2, PSMD2, STIL, TTK and other genes in tumors, while ALDOA gene is downregulated. **(B)** Chromosomal localization analysis of differentially expressed stem cell marker genes, with outer ring showing chromosomal positions, inner red dots representing genes significantly highly expressed in tumors (p<0.05), and central PCA plot displaying sample distribution from seven lung adenocarcinoma transcriptomic datasets after batch correction. **(C)** KEGG pathway enrichment analysis of differentially expressed genes, showing these genes mainly participate in cell cycle, DNA replication, metabolic reprogramming and other key biological pathways. **(D)** GO functional enrichment analysis of differentially expressed genes, including biological processes (BP, red), cellular components (CC, blue), and molecular functions (MF, green), revealing gene functions in cell division and proliferation regulation.

To gain deeper insights into the expression changes of stem cell marker genes in LUAD, we performed systematic differential expression analysis of stem cell-related genes between GTEx normal lung tissues and TCGA LUAD tissues (**Figure 2A**). The heatmap results revealed complex expression regulatory patterns of stem cell marker genes in tumor tissues. Among these, key genes including TMEM106C, ECT2, PSMD2, STIL, and TTK showed significant upregulation in tumor tissues, and their high expression may be closely associated with abnormal activation of tumor stem cells, cell cycle regulation, and enhanced self-renewal capacity. Notably, the ALDOA gene exhibited downregulation in tumor tissues, suggesting that different stem cell marker genes may play distinct regulatory roles during tumorigenesis. Further analysis of the chromosomal localization characteristics of these differentially expressed stem cell marker genes (**Figure 2B**) revealed that these genes are widely distributed across various chromosomes, with genes significantly highly expressed in tumors (p<0.05, marked by red dots) mainly concentrated in specific chromosomal regions. The central PCA analysis plot displays the sample distribution pattern from seven LUAD transcriptomic datasets after batch effect correction, demonstrating good consistency in stem cell marker gene expression patterns across different datasets, providing a reliable data foundation for subsequent functional analysis. To elucidate the biological functions of these differentially expressed stem cell marker genes, we performed KEGG pathway enrichment analysis (**Figure 2C**). The results showed that differentially expressed genes were mainly enriched in core pathways related to cell proliferation regulation, including cell cycle, DNA replication, and p53 signaling pathway, while also being significantly enriched in metabolic reprogramming-related pathways such as glycolysis/gluconeogenesis and HIF-1 signaling pathway. The enrichment of these pathways suggests that abnormal expression of stem cell marker genes may affect tumor stem cell function maintenance by simultaneously regulating cell proliferation and metabolic reprogramming. GO functional enrichment analysis further revealed the specific functional characteristics of these genes (**Figure 2D**). In terms of biological processes, differentially expressed genes mainly participate in nuclear division, cell division, and other cell proliferation-related processes; regarding cellular components, these genes are primarily localized to chromosomes, centromeric regions, and other cellular structures closely related to cell division; in terms of molecular functions, differentially expressed genes mainly possess ATP-dependent DNA activity, microtubule motor activity, and other functions. These functional enrichment results further confirm the important role of stem cell marker genes in regulating cell division and proliferation, which are core stem cell functions.

### Construction and validation of stem cell prognostic model

**Figure 3:**
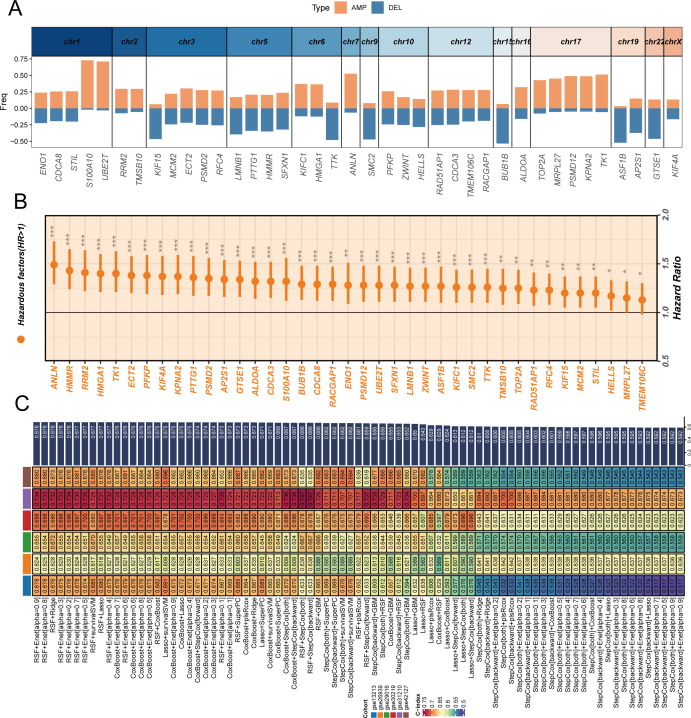
Construction and validation of stem cell prognostic model through genomic analysis and machine learning approaches. **(A)** Chromosomal distribution and copy number variation characteristics of differentially expressed stem cell marker genes, with orange bars representing amplifications (AMP) and blue bars representing deletions (DEL) across different chromosomes, showing that genes like S100A10, UBE2T, and ANLN are predominantly amplified. **(B)** Forest plot showing univariate Cox regression analysis results of stem cell marker genes in TCGA cohort, with hazard ratios and confidence intervals indicating prognostic significance of individual genes. **(C)** Heatmap displaying C-index values of various machine learning algorithm combinations across TCGA training cohort and six GEO validation cohorts, with color intensity representing model performance from blue (low C-index) to red (high C-index). The optimal model CoxBoost+Enet[alpha=0.7] was selected as SCPM (Stem Cell Prognostic Model) based on comprehensive consideration of gene number and C-index performance. *P < 0.05, **P < 0.01, ***P < 0.001.

To deeply explore the genomic characteristics of stem cell marker genes and construct a reliable prognostic prediction model, we first analyzed the chromosomal distribution and copy number variation characteristics of these differentially expressed genes (**Figure 3A**). The results revealed that stem cell marker genes are widely distributed across various chromosomes, with amplification (AMP, orange) and deletion (DEL, blue) events showing specific patterns in different chromosomal regions. Notably, multiple key genes such as S100A10, UBE2T, and ANLN predominantly exhibited gene amplification, while some genes tended to undergo deletion events. This copy number variation pattern may be closely related to the expression changes and functional abnormalities of these genes in tumors. Through univariate Cox regression analysis in the TCGA cohort (**Figure 3B**), we found that all stem cell marker genes had hazard ratios (HR) greater than 1, indicating that high expression of these genes was consistently associated with poor prognosis in LUAD patients, providing a foundation for prognostic model construction. Based on these prognostically valuable stem cell marker genes, we employed a random combination strategy of multiple machine learning algorithms to construct prognostic prediction models (**Figure 3C**). By systematically comparing the performance of different algorithm combinations in the TCGA training cohort and six GEO validation cohorts, we used the C-index as an evaluation metric to measure model prediction accuracy. The heatmap displays C-index values of various algorithm combinations across different cohorts, with colors ranging from blue to red representing C-index values from low to high. Considering both the number of genes included in the model and the C-index performance comprehensively, we ultimately selected the CoxBoost+Enet[alpha=0.7] combination as the optimal model and named it SCPM (Stem Cell Prognostic Model). This model not only demonstrated good predictive performance in the training cohort but also showed stable prognostic prediction capability across multiple independent validation cohorts.

### Validation of SCPM prognostic performance and immunotherapy prediction value

**Figure 4:**
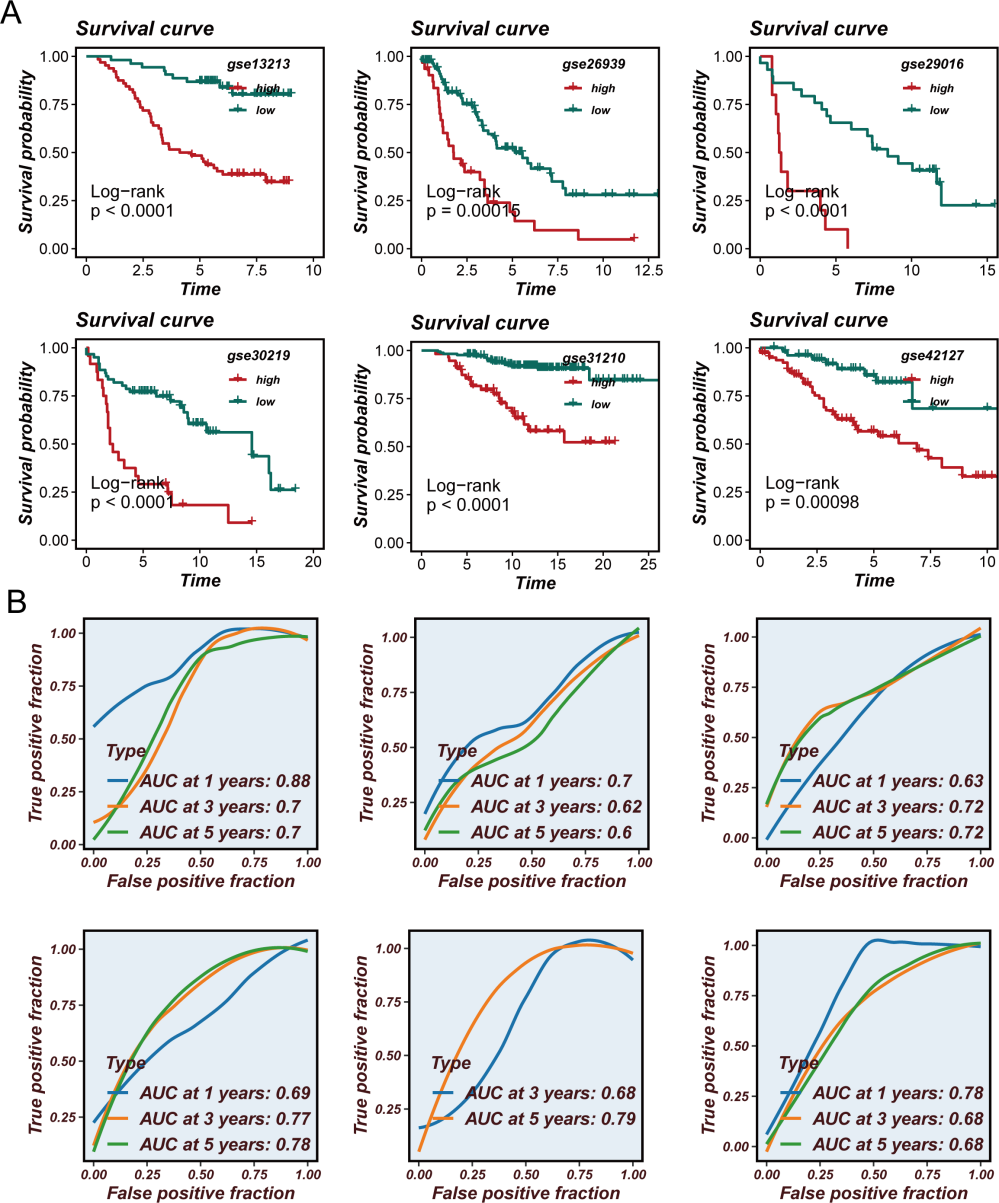
Validation of SCPM prognostic performance across independent GEO cohorts. **(A)** Kaplan-Meier survival curves showing prognostic stratification capability of SCPM across six independent validation cohorts (GSE13213, GSE26939, GSE29016, GSE30219, GSE31210, and GSE42127), with significant survival differences between high-SCPM (red) and low-SCPM (green) groups (all p < 0.05). **(B)** Time-dependent ROC curves demonstrating predictive accuracy of SCPM for 1-, 3-, and 5-year survival across validation cohorts, with AUC values consistently above 0.6.

**Figure 5:**
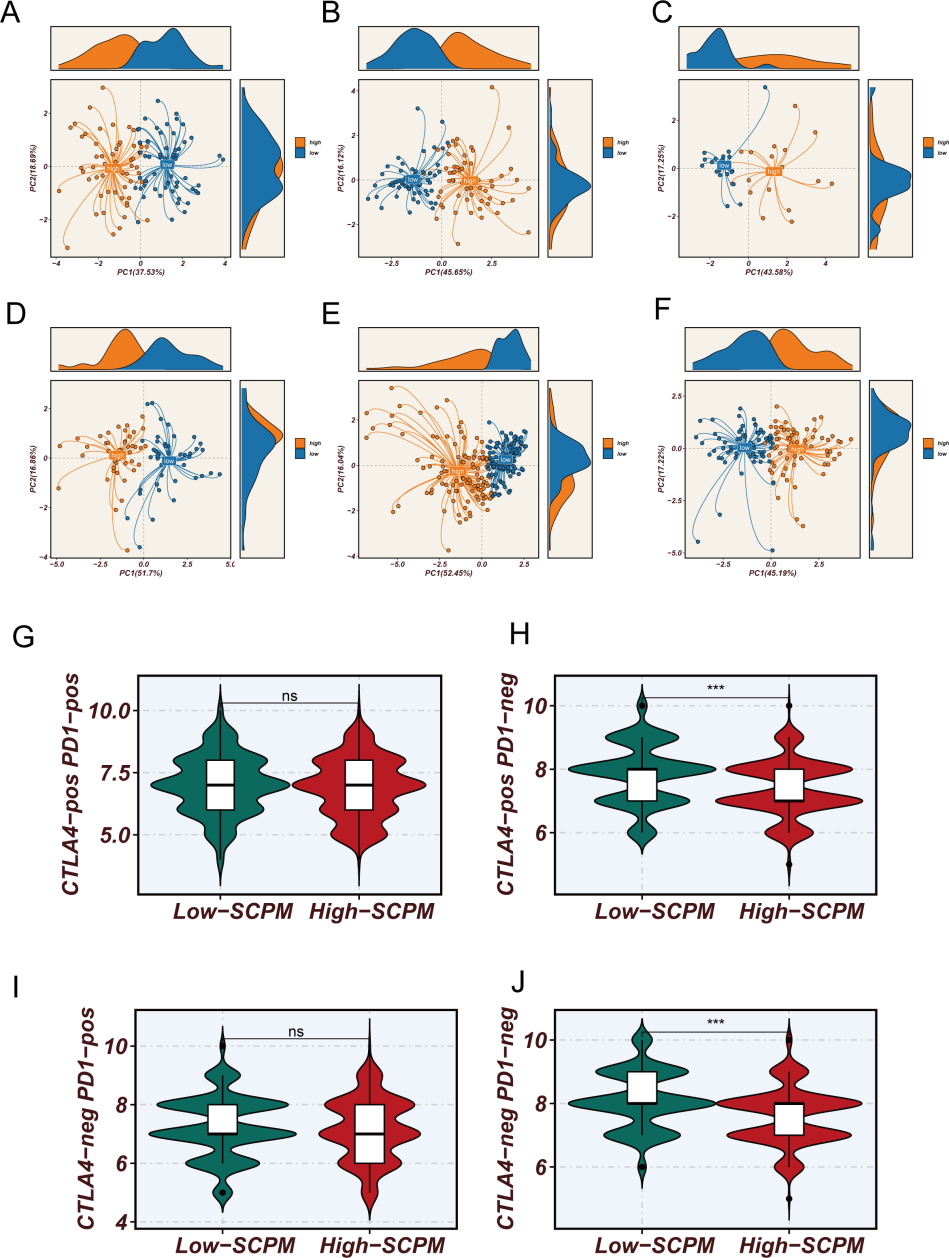
Molecular characterization validation and immunotherapy prediction value of SCPM model. **(A–F)** Principal component analysis based on 7 SCPM signature gene expression profiles showing clear separation between high-SCPM (orange) and low-SCPM (blue) groups across six validation cohorts, validating the molecular biological foundation and cross-cohort consistency of the model. **(G–J)** Comparison of TCIA scores between high-SCPM and low-SCPM groups, showing significantly higher immunotherapy sensitivity scores in low-SCPM group patients for CTLA4-pos PD1-neg **(H)** and CTLA4-neg PD1-pos **(J)** subtypes (***p < 0.001), while differences were not statistically significant for CTLA4-pos PD1-pos **(G)** and CTLA4-neg PD1-neg **(I)** subtypes (ns).

To validate the robustness and generalizability of the SCPM model, we first confirmed its performance in the TCGA training cohort (**Supplementary Figure S1**). Patients were stratified into high-SCPM and low-SCPM groups based on SCPM scores, demonstrating excellent prognostic stratification with significant survival differences (Log-rank p < 0.0001) (**Supplementary Figure S1A**). Time-dependent ROC analysis showed good predictive accuracy with AUC values of 0.68, 0.68, and 0.64 for 1-, 3-, and 5-year survival predictions, respectively (**Supplementary Figure S1B**). Principal component analysis based on SCPM signature genes revealed distinct clustering patterns between high-SCPM and low-SCPM groups (**Supplementary Figure S1C**), indicating that the model effectively captures biological differences related to stem cell characteristics. Subsequently, we systematically evaluated SCPM prognostic performance across six independent GEO validation cohorts (**Figure 4**). Kaplan-Meier survival analysis consistently demonstrated significant prognostic stratification capability across all validation datasets (**Figure 4A**). SCPM score-based stratification effectively distinguished patients into prognostically distinct subgroups, further confirming the robust discriminatory power of the SCPM scoring system. Time-dependent ROC analysis (**Figure 4B**) further validated the predictive accuracy of SCPM across multiple time points, demonstrating good predictive performance in all validation cohorts. It should be noted that, since most patients in the GSE31210 cohort had survival times exceeding one year, it was not possible to calculate the ROC curve and AUC value at the 1-year time point for this cohort. To gain deeper insights into the biological foundation of the SCPM model, we performed principal component analysis based on the expression profiles of 7 SCPM signature genes across six validation cohorts (**Figures 5A–F**). Results showed that patient samples could be clearly separated into two distinct clusters based on SCPM signature gene expression patterns across all validation cohorts, with high-SCPM (orange) and low-SCPM (blue) groups showing clear separation in principal component space, further validating the molecular biological rationale and cross-cohort consistency of the SCPM model.

Considering the close relationship between stem cell characteristics and the tumor immune microenvironment, we further analyzed the association between SCPM scores and immunotherapy sensitivity (**Figures 5G–J**). Using The Cancer Immunome Atlas (TCIA) scoring system, we systematically evaluated the predicted responsiveness of different SCPM groups to immune checkpoint inhibitors across multiple immunotherapy subtypes. The results showed that the low-SCPM group demonstrated better performance in several immunotherapy response prediction metrics. Further subgroup analysis revealed that this benefit was mainly observed in patients receiving CTLA-4 inhibitors or double-negative immunotherapy regimens, while no statistically significant differences were found in other immunotherapy subtypes. These findings suggest the potential value and limitations of the SCPM model in guiding precision immunotherapy, indicating that patients in the low-SCPM group may be more suitable for specific types of immunotherapeutic strategies.

### Validation of SCPM model in immunotherapy cohorts

**Figure 6:**
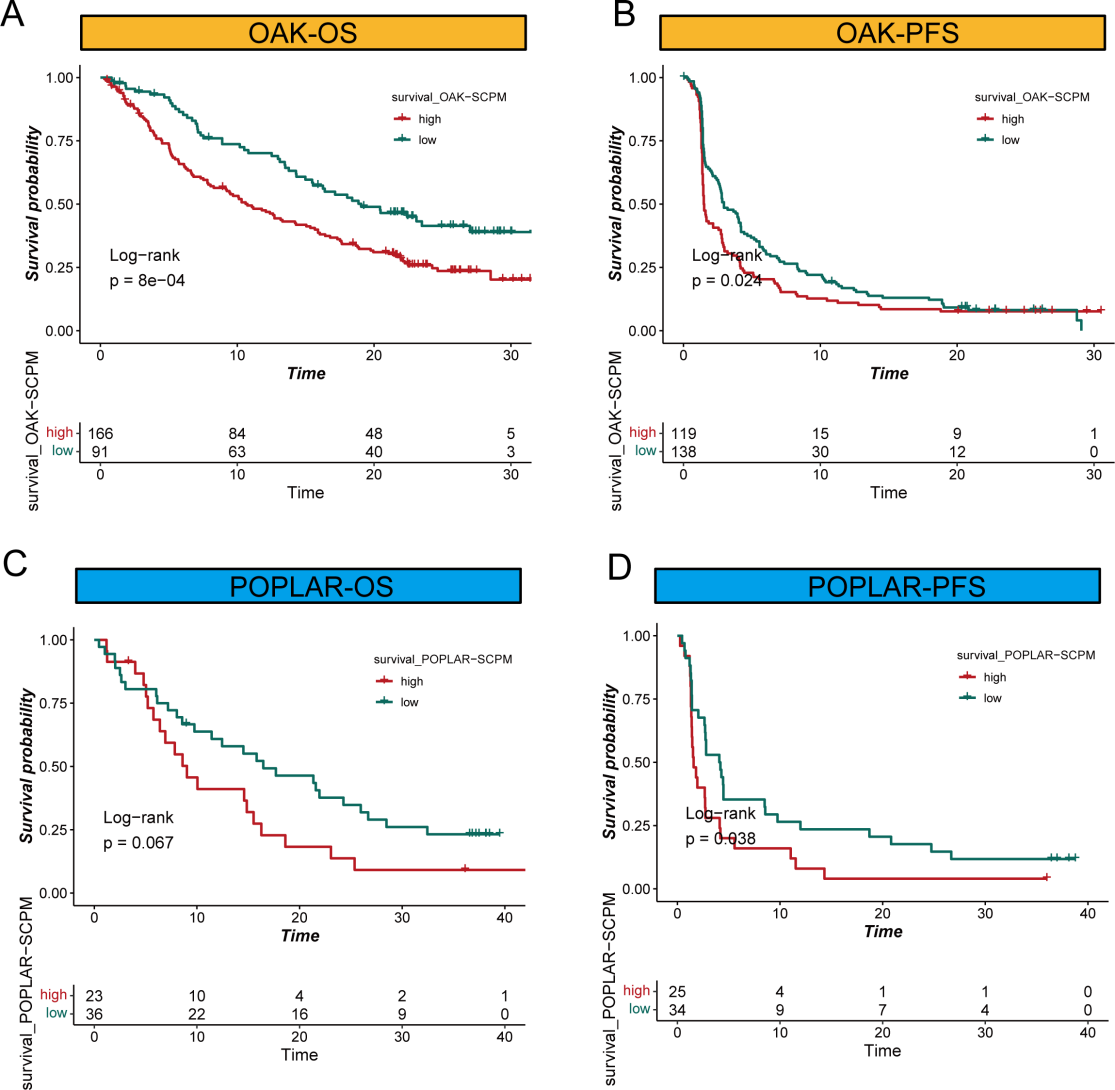
SCPM prognostic performance in immunotherapy cohorts. **(A, B)** Kaplan-Meier survival curves in the OAK immunotherapy cohort showing significant prognostic stratification for both overall survival (OS, Log-rank p = 8e-04) and progression-free survival (PFS, Log-rank p = 0.024) between high-SCPM (red) and low-SCPM (green) groups. **(C, D)** Survival analysis in the POPLAR immunotherapy cohort demonstrating prognostic stratification for OS (Log-rank p = 0.067) and PFS (Log-rank p = 0.038) between high-SCPM and low-SCPM groups.

To further validate the clinical utility of SCPM in the context of immunotherapy, we evaluated its prognostic performance across three independent immunotherapy cohorts (**Figure 6**, **Supplementary Figure S2**). In the OAK and POPLAR clinical trial cohorts, SCPM demonstrated good prognostic stratification capability. In the OAK cohort, the high-SCPM group showed significantly worse outcomes compared to the low-SCPM group for both OS and PFS (Log-rank p = 8e-04 and p = 0.024, respectively) (**Figures 6A, B**). In the POPLAR cohort, OS difference approached statistical significance (Log-rank p = 0.067), while PFS difference reached statistical significance (Log-rank p = 0.038) (**Figures 6C, D**). Additional validation was performed in the SU2C immunotherapy cohort (**Supplementary Figure S2**). The SCPM model maintained its prognostic capability with significant OS stratification (Log-rank p = 0.028) (**Supplementary Figure S2A**), while PFS stratification showed a similar trend but did not reach statistical significance (Log-rank p = 0.25) (**Supplementary Figure S2B**). These results across multiple independent immunotherapy cohorts consistently support the prognostic value of SCPM and its potential utility in guiding immunotherapy treatment decisions.

### SCPM-associated immune microenvironment characteristics

**Figure 7:**
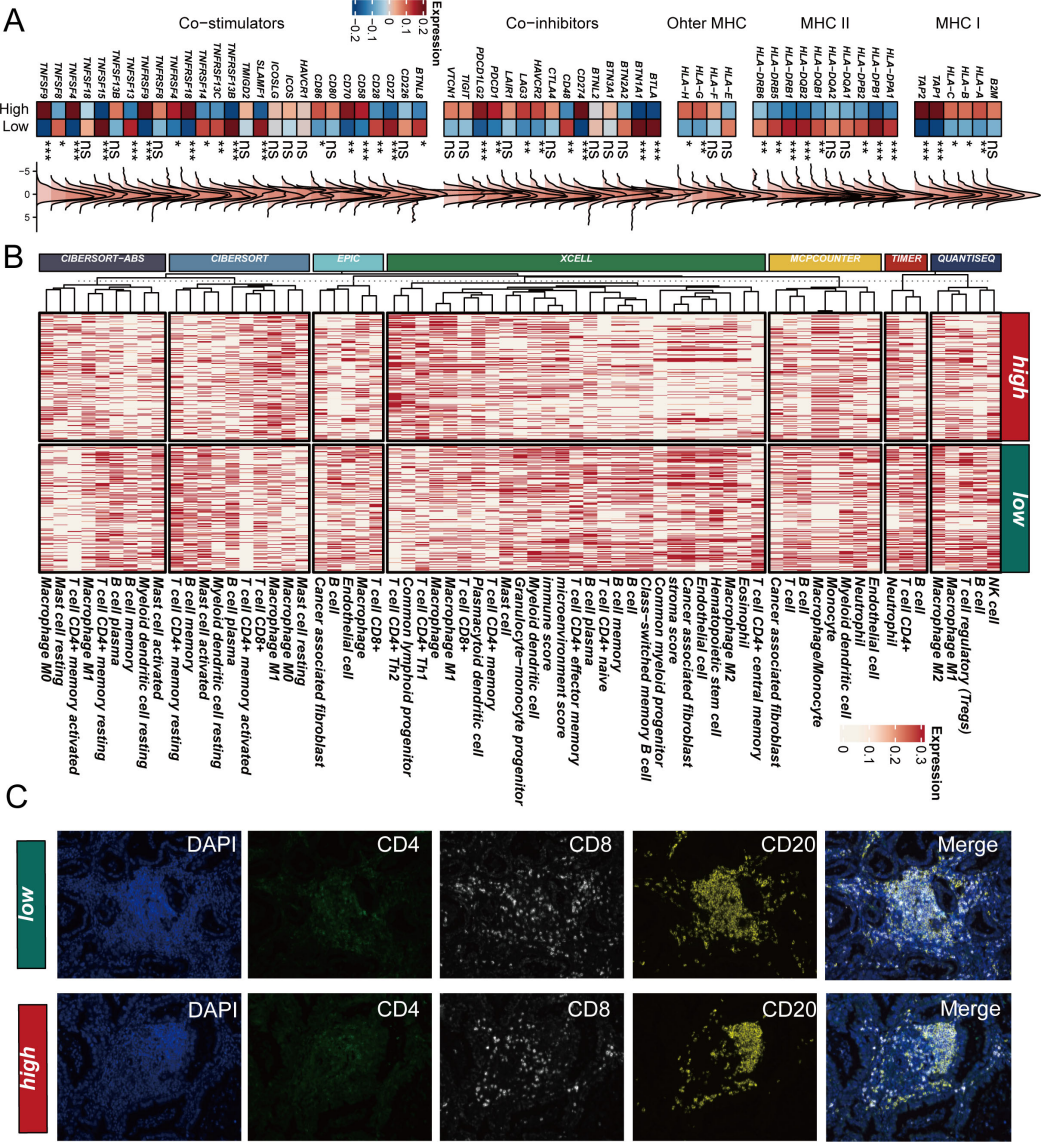
SCPM-associated immune microenvironment characteristics. **(A)** Heatmap showing differential expression of immune-related genes between high-SCPM and low-SCPM groups, including co-stimulators, co-inhibitors, and MHC molecules. High-SCPM group shows reduced HLA expression but elevated immune checkpoint molecules (PDCD1, CD274, TIGIT). **(B)** Immune cell infiltration analysis using seven computational algorithms (CIBERSORT-ABS, CIBERSORT, EPIC, XCELL, MCP-COUNTER, TIMER, QUANTISEQ) demonstrates consistently higher immune infiltration in low-SCPM group (green) compared to high-SCPM group (red). **(C)** Multiplex immunofluorescence validation in clinical samples showing enhanced CD8+ T cell and CD20+ B cell infiltration in low-SCPM tumors compared to high-SCPM tumors. DAPI (blue), CD4 (green), CD8 (white), CD20 (yellow), and merged images are shown for representative low-SCPM and high-SCPM cases.

To elucidate the underlying mechanisms of SCPM’s prognostic and immunotherapeutic predictive value, we comprehensively analyzed the immune microenvironment characteristics between high-SCPM and low-SCPM groups. Analysis of immune-related gene expression revealed distinct patterns between the two groups (**Figure 7A**). Notably, the high-SCPM group showed reduced expression of HLA class genes, particularly MHC class I and II molecules, indicating potential immune evasion mechanisms. Conversely, the high-SCPM group exhibited elevated expression of immune checkpoint molecules including PDCD1, CD274, and TIGIT, suggesting an immunosuppressive microenvironment despite the presence of checkpoint molecules. To further characterize immune infiltration patterns, we employed seven different computational algorithms to evaluate immune cell infiltration levels across SCPM groups (**Figure 7B**). Consistent across multiple algorithms including CIBERSORT-ABS, CIBERSORT, EPIC, XCELL, MCP-COUNTER, TIMER, and QUANTISEQ, the low-SCPM group demonstrated significantly higher levels of immune cell infiltration compared to the high-SCPM group. This enhanced immune infiltration in low-SCPM tumors suggests a more immunologically “hot” tumor microenvironment that may be more responsive to immunotherapy. To validate these computational findings, we performed multiplex immunofluorescence staining on clinical samples from our institutional cohort (**Figure 7C**). RNA-seq data from our internal cohort was used to stratify patients into high-SCPM and low-SCPM groups, followed by immunofluorescence validation using CD4, CD8, and CD20 markers. The results confirmed our computational predictions, showing that low-SCPM tumors had significantly enhanced infiltration of CD8+ T cells and CD20+ B cells compared to high-SCPM tumors. This experimental validation strongly supports the association between low SCPM scores and increased immune cell infiltration, providing a mechanistic basis for the superior immunotherapy responsiveness observed in low-SCPM patients.

## Discussion

In this comprehensive study, we developed and validated SCPM, a novel stem cell-based prognostic model for LUAD that demonstrates robust predictive capability across multiple independent cohorts and provides valuable insights for immunotherapy decision-making. Our findings reveal the critical role of stem cell characteristics in determining patient prognosis and treatment response, offering new perspectives for precision medicine in lung cancer. Single-cell RNA sequencing analysis revealed the complex cellular ecosystem within the LUAD microenvironment, with stem cell marker genes showing distinct expression patterns across different cell types. We identified widespread mutations in stem cell-related genes, particularly the high frequency of KRAS mutations (29%), which aligns with previous studies emphasizing the central role of KRAS in cancer stem cell biology (36, 37). The prevalence of KRAS mutations in our cohort is consistent with its established function in maintaining stem cell self-renewal and promoting oncogenic transformation. Similarly, the frequent mutations in KEAP1 suggest that oxidative stress response is a critical pathway disrupted in LUAD stem cells (38).

Comprehensive differential expression analysis between normal and tumor tissues revealed complex regulatory patterns of stem cell marker genes, with key genes such as TMEM106C, ECT2, PSMD2, STIL, and TTK showing significant upregulation in tumor tissues. These findings are consistent with the cancer stem cell hypothesis, which posits that a small subset of cells with stem-like properties drives tumor initiation, progression, and therapeutic resistance (39). The enrichment of differentially expressed genes in cell cycle regulation, DNA replication, and metabolic reprogramming pathways suggests that stem cell characteristics are intimately linked to fundamental cellular processes that drive tumorigenesis. Notably, the downregulation of ALDOA in tumor tissues highlights the metabolic reprogramming that occurs in cancer stem cells, potentially reflecting their unique metabolic dependencies compared to bulk tumor cells. Chromosomal distribution analysis revealed that stem cell marker genes are subject to widespread copy number alterations, with amplification events predominating in genes such as S100A10, UBE2T, and ANLN, suggesting that genomic instability contributes to the dysregulation of stem cell programs in LUAD.

The application of multiple machine learning algorithms in cancer biomarker development has advanced rapidly (40, 41). The consistent performance of SCPM across seven independent cohorts, including both transcriptomic datasets and immunotherapy cohorts, demonstrates its robustness and clinical applicability. The superior prognostic stratification achieved by SCPM, particularly in the context of immunotherapy, suggests that stem cell characteristics serve as important determinants of treatment response. The association between low SCPM scores and improved immunotherapy outcomes may be explained by the enhanced immune cell infiltration observed in low-SCPM tumors. We found that low-SCPM tumors exhibit higher levels of CD8+ T cells and CD20+ B cells, confirmed by both computational analysis and experimental validation, providing mechanistic insights into why these patients respond better to immune checkpoint inhibitors. This is consistent with emerging evidence that cancer stem cells can modulate the immune microenvironment and influence immunotherapy efficacy (42).

Immune microenvironment analysis revealed striking differences between high-SCPM and low-SCPM groups, with high-SCPM tumors characterized by reduced HLA expression and elevated immune checkpoint molecule expression, suggesting an immunosuppressive phenotype despite the presence of immune activation markers. This paradoxical pattern may reflect the complex interplay between stem cell characteristics and immune evasion mechanisms. The reduced expression of MHC class I and II molecules in high-SCPM tumors indicates impaired antigen presentation capacity, which may limit the effectiveness of T cell-mediated immune responses. The clinical implications of our findings extend beyond prognostic stratification to treatment selection, as the SCPM model may help identify patients who are most likely to benefit from immunotherapy. Future studies should focus on validating these findings in prospective clinical trials and exploring the potential for targeting stem cell pathways in combination with immunotherapy to improve outcomes for high-SCPM patients.

In this study, we successfully developed and validated SCPM, a novel prognostic prediction tool for LUAD based on stem cell marker genes. Through integrated multi-omics analysis and advanced machine learning algorithms, we demonstrated that SCPM provides robust prognostic stratification across multiple independent cohorts, with particularly strong performance in predicting responses to immunotherapy. Our findings reveal that patients with low SCPM scores exhibit higher levels of immune cell infiltration and respond more favorably to immunotherapy, offering valuable prognostic assessment and guidance for clinical decision-making. The SCPM model holds significant promise for clinical application, enabling the identification of patient subgroups most likely to benefit from immunotherapy and advancing the precision treatment of LUAD.

## Data Availability

The original contributions presented in the study are included in the article/**Supplementary Material**. Further inquiries can be directed to the corresponding authors.

## Supplementary material

Supplementary Figure 1 SCPM performance in TCGA training cohort. **(A)** Kaplan-Meier survival curve showing significant prognostic stratification between high-SCPM and low-SCPM groups (Log-rank p < 0.0001). **(B)** Time-dependent ROC curves for 1-, 3-, and 5-year survival predictions with AUC values of 0.68, 0.68, and 0.64, respectively. **(C)** Principal component analysis showing distinct clustering patterns between high-SCPM (orange) and low-SCPM (blue) groups based on SCPM signature genes.

Supplementary Figure 2 SCPM prognostic performance in SU2C immunotherapy cohort. **(A, B)** Kaplan-Meier survival curves showing significant OS stratification (Log-rank p = 0.028) and PFS trend (Log-rank p = 0.25) between high-SCPM (red) and low-SCPM (green) groups in the SU2C immunotherapy cohort.
