# Supplementary figures and images for: Single-cell and multi-omics analysis reveals the role of stem cells in prognosis and immunotherapy of lung adenocarcinoma patients
Source: Front Immunol. 2025 Jul 22;16:1634830. doi: 10.3389/fimmu.2025.1634830 (PMC12321537; doi:10.3389/fimmu.2025.1634830)

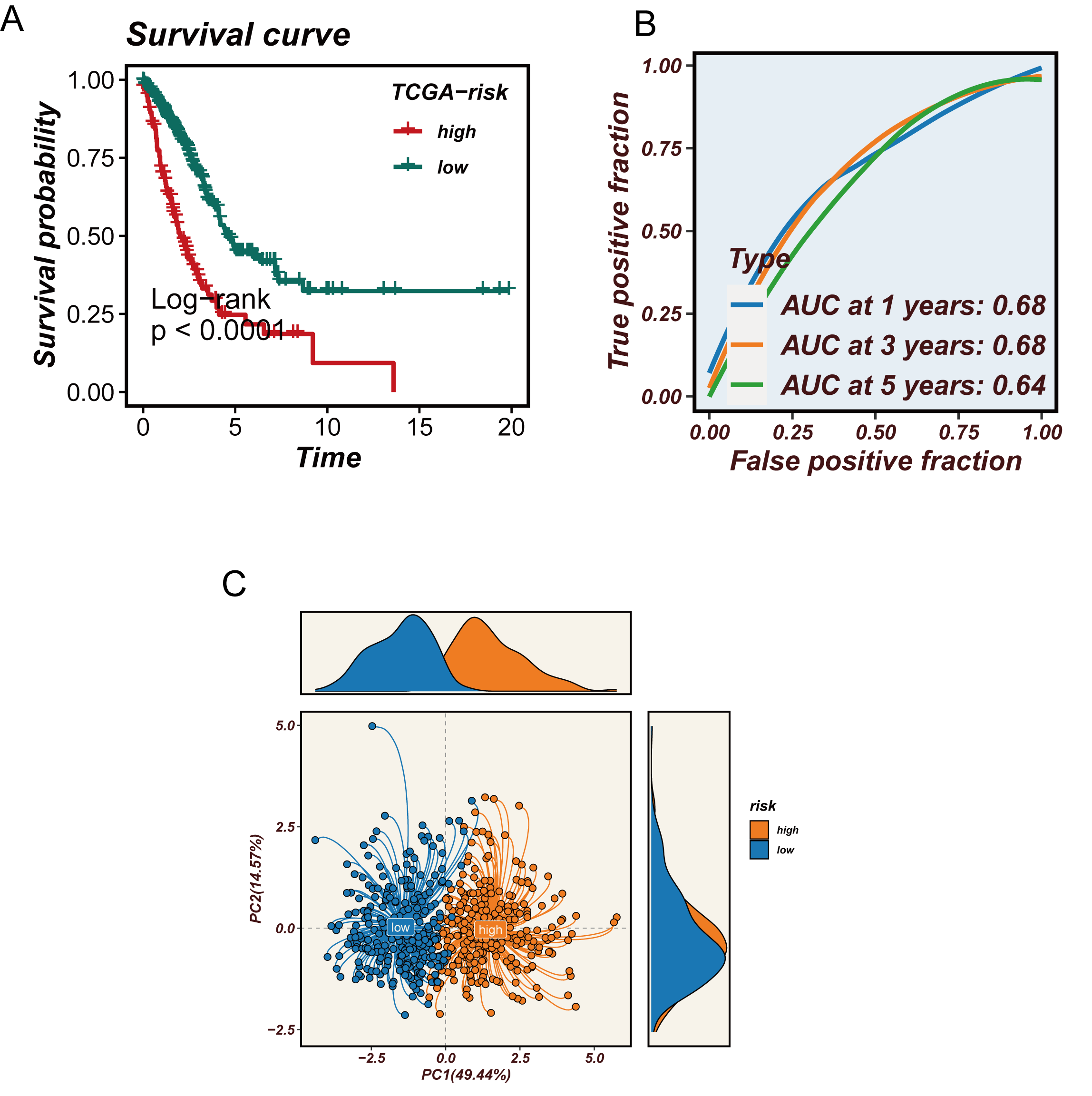

Supplement: Supplementary Figure 1 — SCPM performance in TCGA training cohort. (A) Kaplan-Meier survival curve showing significant prognostic stratification between high-SCPM and low-SCPM groups (Log-rank p < 0.0001). (B) Time-dependent ROC curves for 1-, 3-, and 5-year survival predictions with AUC values of 0.68, 0.68, and 0.64, respectively. (C) Principal component analysis showing distinct clustering patterns between high-SCPM (orange) and low-SCPM (blue) groups based on SCPM signature genes. [file Image1.tif]

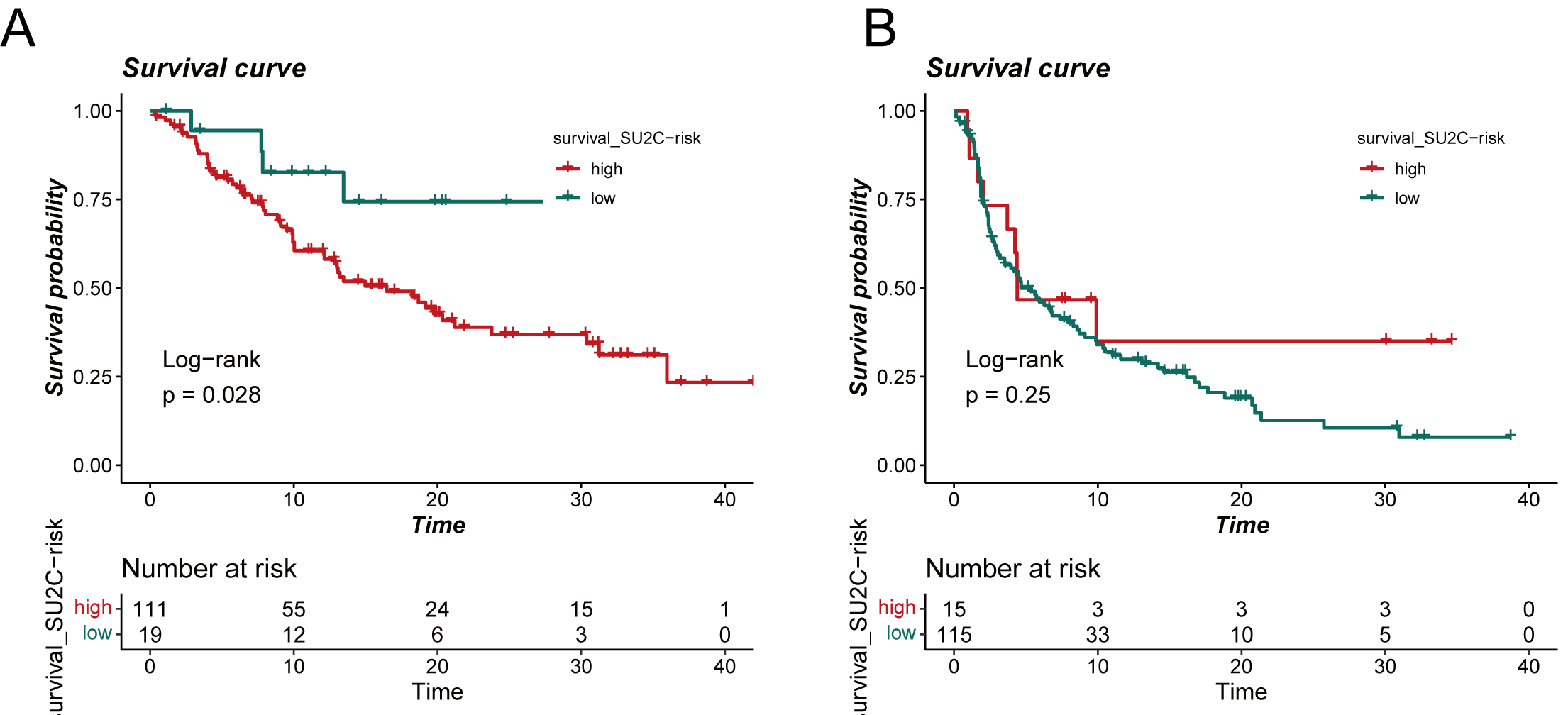

Supplement: Supplementary Figure 2 — SCPM prognostic performance in SU2C immunotherapy cohort. (A, B) Kaplan-Meier survival curves showing significant OS stratification (Log-rank p = 0.028) and PFS trend (Log-rank p = 0.25) between high-SCPM (red) and low-SCPM (green) groups in the SU2C immunotherapy cohort. [file Image2.tif]
